# Supplementary material for: The recovery of European freshwater biodiversity has come to a halt
Source: Nature. 2023 Aug 9;620(7974):582–8. doi: 10.1038/s41586-023-06400-1 (PMC10432276; doi:10.1038/s41586-023-06400-1)
Supplement: Supplementary file 1 — Supplementary equation (1) and Supplementary Tables 1–6 [file 41586_2023_6400_MOESM1_ESM.pdf]

nature portfolio

https://doi.org/10.1038/s41586-023-06400-1

Supplementary information

The recovery of European freshwater biodiversity has come to a halt

In the format provided by the authors and unedited

Supplementary Information for:

The recovery of European freshwater biodiversity has come to a halt

Authors

Peter Haase, Diana E. Bowler, Nathan J. Baker, Núria Bonada, Sami Domisch, Jaime R. Garcia Marquez, Jani Heino, Daniel Hering, Sonja C. Jähnig, Astrid Schmidt-Kloiber, Rachel Stubbington, Florian Altermatt, Mario Álvarez-Cabria, Giuseppe Amatulli, David Angeler, Gaït Archambaud, Iñaki Arrate Jorrín, Thomas Aspin, Iker Azpiroz, Iñaki Bañares, José Barquín Ortiz, Christian L. Bodin, Luca Bonacina, Roberta Bottarin, Miguel Cañedo-Argüelles, Zoltán Csabai, Thibault Datry, Elvira de Eyto, Alain Dohet, Gerald Dörflinger, Emma Drohan, Knut A. Eikland, Judy England, Tor E. Eriksen, Vesela Evtimova, Maria J. Feio, Martial Ferréol, Mathieu Floury, Maxence Forcellini, Marie Anne Forio, Riccardo Fornaroli, Nikolai Friberg, Jean-François Fruget, Galia Georgieva, Peter Goethals, Manuel A.S. Graça, Wolfram Graf, Kaisa-Leena Huttunen, Thomas C. Jensen, Richard K. Johnson, J. Iwan Jones, Jens Kiesel, Lenka Kuglerová, Aitor Larrañaga, Patrick Leitner, Lionel L'Hoste, Marie-Helène Lizée, Armin W. Lorenz, Anthony Maire, Jesús Alberto Manzanos Arnaiz, Brendan G. McKie, Andrés Millán, Don Monteith, Timo Muotka, John F. Murphy, Davis Ozolins, Riku Paavola, Petr Paril, Francisco J. Peñas, Francesca Pilotto, Marek Polasek, Jes Jessen Rasmussen, Manu Rubio, David Sánchez-Fernández, Leonard Sandin, Ralf B. Schäfer, Alberto Scotti, Longzhu Q. Shen, Agnija Skuja, Stefan Stoll, Michal Straka, Henn Timm, Violeta G. Tyufekchieva, Iakovos Tziortzis, Yordan Uzunov, Gea H. van der Lee, Rudy Vannevel, Emilia Varadinova, Gábor Várbíró, Gaute Velle, Piet F.M. Verdonschot, Ralf C.M. Verdonschot, Yanka Vidinova, Peter Wiberg-Larsen, Ellen A.R. Welti

Equation 1. Calculation of dam impact scores.

Dam impact scores were calculated using only upstream connected dams within 100 km of a site as:

$$\sum_{i=1}^{n} \frac{100 - d_i}{100}$$

where *n* represents the number of dams, *i* denotes a given dam, and *d* is the distance (km) of the dam from a site.

Supplementary Table 1: Citations for stressors and legislation included in the timeline in Figure 1a.

Year	Type	Event	Citation
1950s–1970s	Stressor	Start of the Great Acceleration	Steffen, W., Broadgate, W., Deutsch, L., Gaffney, O. & Ludwig, C. The trajectory of the Anthropocene: The Great Acceleration. *Anthr. Rev.* **2**, 81–98 (2015).
1980	Stressor	Peak acidification	Grennfelt, P. et al. Acid rain and air pollution: 50 years of progress in environmental science and policy. *Ambio* **49**, 849–864 (2020).
1988	Stressor	Peak EU use of N and P fertilisers	Nitrogen (N): van Grinsven, H. J. M. et al. Losses of ammonia and nitrate from agriculture and their effect on nitrogen recovery in the European Union and the United States between 1900 and 2050. *J. Environ. Qual.* **44**, 356–367 (2015).<br><br>Phosphorus (P): Schoumans, O. F., Bouraoui, F., Kabbe, C., Oenema, O. & van Dijk, K. C. Phosphorus management in Europe in a changing world. *Ambio* **44**, 180–192 (2015).
1996	Stressor	Peak non-native species introduction	Seebens, H. et al. No saturation in the accumulation of alien species worldwide. *Nat. Commun.* **8**, 14435 (2017).
2003	Stressor	Hottest European summer in 500 years	Stott, P. A., Stone, D. A. & Allen, M. R. Human contribution to the European heatwave of 2003. *Nature* **432**, 610–614 (2004).
2005	Stressor	New neonicotinoid insecticides authorised in Europe	Simon-Delso, N. *et al.* Systemic insecticides (neonicotinoids and fipronil): trends, uses, mode of action and metabolites. *Environ. Sci. Pollut. Res.* **22**, 5–34 (2015).

2021	Stressor	Hottest European summer	Copernicus Climate Change Service (C3S). Copernicus: Globally, the seven hottest years on record were the last seven; carbon dioxide and methane concentrations continue to rise. https://climate.copernicus.eu/copernicus-globally-seven-hottest-years-record-were-last-seven (2022).
1979	Legislation	UN/ECE LRTAP Sulphur Protocols	CLRTAP 1979
1991	Legislation	EU Urban Waste Water Treatment Directive	European Commission 1991
2000	Legislation	EU Water Framework Directive	European Commission 2000
2016	Legislation	Paris Agreement	UNFCCC 2015
2018	Legislation	EU Strategy for Plastics in a Circular Economy	European Commission 2018

Supplementary Table 2: Time series locations, durations, and site characteristics.

Due to the large size of this table, we have uploaded it separately as an Excel document to our online repository:

https://github.com/Ewelti/EuroAquaticMacroInverts/blob/main/plots/Supplementary%20Table%202.xlsx

Supplementary Table 3: Number of sites sampled per year and country.

Year	Austria	Belgium	Bulgaria	Cyprus	Czech Republic	Denmark	Estonia	Finland	France	Germany	Hungary	Ireland	Italy	Latvia	Luxemburg	Netherlands	Norway	Portugal	Spain	Sweden	Switzerland	UK	Total
**1968**										1													1
**1969**										3													3
**1971**										21													21
**1972**										35													35
**1973**										39													39
**1974**										12													12
**1975**										3													3
**1976**										28													28
**1977**										29													29
**1978**										33													33
**1979**			2							23													25
**1980**			2						1	32						1							36
**1981**			2						1	3						10	1						17
**1982**										75						8							83
**1983**			1						1	66						10	2						80
**1984**									1	50						14							65
**1985**			1						1	87						15	1						105
**1986**			4						1	80				1		15	1		4				106
**1987**			4							85				1		15	2		30				137
**1988**			1						1	98				1		16	3						120
**1989**		20	1						1	61				1		12			11				107
**1990**		10	1						1	80				1		15			9				117
**1991**		18	3						1	78						15	3		13				131
**1992**		17				54			90	87						10			13				271
**1993**		27	1			82			80	53						11		2	45				301
**1994**		31				91			85	88						12		2	53			45	407
**1995**		36				95			110	86						13		2	53	1	1	86	483
**1996**		32				83			119	68				2		8		2	95	3		77	489
**1997**		43	2			87			230	49				3		10		2	105	12		95	638
**1998**		47				118			208	56				3		14		2	112	11		114	685
**1999**		23				216			220	87				3		15		2	120	11		88	785
**2000**		43				213		10	199	55				3		20		2	115	14	1	104	779
**2001**		45				210		10	203	54				3		23		2	114	14		62	740
**2002**		42			1	227		10	137	46				3		24		2	139	13		119	763
**2003**		50			1	229	1	10	132	64		16		1		22	35	2	147	16		180	906
**2004**		39				242		10	126	33		16		3		30	49	2	144	34	1	183	912
**2005**		39	3		1	237		10	73	42	45	16		3		30	54	2	144	45		198	942
**2006**		39	1	2		243	1	10	69	24	42	16		3		24	49	2	150	51		251	977
**2007**		30		1		238		10	194	36	68	16		3	20	28	43	2	204	85		264	1242
**2008**	2	15		2		241		10	220	18	77	16		3	20	25	41	2	214	83		276	1265
**2009**	1	9	2	2		237		10	213	12	80	16		3	20	33	50	2	213	82		297	1282
**2010**	1	19	8	2		235	1	10	222	14	76	16		3		29	38	2	208	84		276	1244
**2011**	2	12	7	2		202	1	10	226	13	86	16	2	3	20	20	38	2	195	89	1	304	1251
**2012**		8	5		1	245	9	10	231	13	75	16	2	3	20	35	48	2	228	87	1	289	1328
**2013**	1	21	5		1	247	10	10	231	13	76	16	2	3	20	26	26	2	239	89		359	1397
**2014**	1	19	4		1	231	10	10	198	6	82	16	2	3	20	32	65	2	231	87		323	1343
**2015**	2	8	5		1	237	10		211	8	83	16	2	3	20	31	39	2	229	79	1	314	1301
**2016**	1	19	1	1	1	237	10		193	7	81	16	2		20	33	50	2	193	80		308	1255
**2017**	2	10	4	1		47	10		65	6	71	16	2		20	27	38	2	177	81	1	295	875
**2018**	2	6	4	2		237	10		6	5	14	16	2			26	50	2	118	76	1	268	845
**2019**	1	20	5	1		51	10		6	5	11	13	2			35	39	2	120	57		177	555
**2020**		1				2											47						50

Supplementary Table 4: Definitions and citations for functional diversity, functional niche and functional response metrics.

Term	Explanation	Reference
Functional diversity	The value and range of functional traits of the organisms in a given ecosystem	Tilman, D. Functional diversity, in: Levin, S.A. (Ed.), Encyclopaedia of Biodiversity. Academic Press, pp. 109–121 (2001).
Functional niche	Represents an n-dimensional hypervolume in functional space, whereby the axes of the functional space are functions or processes associated with different functional traits	Rosenfeld, J. S. Functional redundancy in ecology and conservation. Oikos 98, 156–162 (2002).
Functional divergence	A measure of how spread or clumped taxa are in a functional space	
Functional evenness	Distribution of abundances in functional space	Mason, N. W. H., Mouillot, D., Lee, W. G. & Wilson, J. B. Functional richness, functional evenness and functional divergence: the primary components of functional diversity. Oikos 111, 112–118 (2005).
Functional richness	The amount of functional space occupied by all taxa in a given community	
Functional redundancy	The overlap in shared functional traits among taxa	Rosenfeld, J. S. Functional redundancy in ecology and conservation. Oikos 98, 156–162 (2002).
Functional temporal turnover	Change in unique functional traits over time	Villéger, S., Grenouillet, G. & Brosse, S. Decomposing functional β-diversity reveals that low functional β-diversity is driven by low functional turnover in European fish assemblages. Glob. Ecol. Biogeogr. 22, 671–681 (2013).
Rao's quadratic entropy	Functional differences between randomly selected taxa in the niche space	Botta-Dukát, Z. Rao’s quadratic entropy as a measure of functional diversity based on multiple traits. J. Veg. Sci. 16, 533–540 (2005).

Supplementary Table 5: Stream characteristics and environmental drivers included as fixed effects in driver models.

Category	Driver	Unit	Temporal duration	Source	Citation
**Stream**	Flow accumulation	km^2	Point	MERIT HYDRO	Yamazaki, D. et al. MERIT Hydro: a high-resolution global hydrography map based on latest topography dataset. *Water Resour. Res.* **55**, 5053–5073 (2019).
	Elevation	m	Point	MERIT HYDRO	
	Slope	Downstream elevation difference divided by distance	Point	90 m network	Amatulli, G. et al. Hydrography90m: a new high-resolution global hydrographic dataset. *Earth Syst. Sci. Data* Preprint, 1–43 (2022). doi.org/10.5194/essd-2022-9
	Strahler stream order	Number of streams joined	Point	90 m network	
**Land cover**	Cropland	% of accumulated area	1992–2018 (annual)	ESA CCI Land Cover	European Space Agency (ESA). Land Cover CCI Product User Guide Version 2. maps.elie.ucl.ac.be/CCI/viewer/download/ESACCI-LC-Ph2-PUGv2_2.0.pdf (2017).
	Urban	% of accumulated area	1992–2018 (annual)	ESA CCI Land Cover	
**Dam**	Dam impact score	Weighted sum of the inverse distance of each connected dam	Point	GRAND v.1.3 (2019)	Lehner, B. et al. High-resolution mapping of the world's reservoirs and dams for sustainable river-flow management. *Front. Ecol. Environ.* **9**, 494–502 (2011).

**Climate**	Precipitation	mm	1958–2019 (monthly)	Terra-Climate	Abatzoglou, J. T., Dobrowski, S. Z., Parks, S. A. & Hegewisch, K. C. TerraClimate, a high-resolution global dataset of monthly climate and climatic water balance from 1958–2015. *Sci. Data* **5**, 170191 (2018).
	Maximum temperature	°C	1958–2019 (monthly)	Terra-Climate	

Supplementary Table 6: Number of time series included in each window of the moving window by country. Windows with ≥250 sites and ≥8 countries were included in the analysis and are shown in yellow. Time series were included within a given window when they had ≥6 sampling years within the window. Two countries from which data were included in other analyses—Cyprus and Switzerland—were not included in any possible window due to gaps in sampling years.

Start Year	End Year	Austria	Belgium	Bulgaria	Cyprus	Denmark	Estonia	Finland	France	Germany	Hungary	Ireland	Italy	Latvia	Luxembourg	Netherlands	Norway	Portugal	Spain	Sweden	United Kingdom	included in moving window?	total	country count
1968	1977									4												no	4	1
1969	1978									14												no	14	1
1970	1979									19												no	19	1
1971	1980									25												no	25	1
1972	1981									22												no	22	1
1973	1982									25												no	25	1
1974	1983									30												no	30	1
1975	1984									32												no	32	1
1976	1985									33												no	33	1
1977	1986								1	31						4						no	36	3
1978	1987								1	31						6						no	38	3
1979	1988								1	35						11	1					no	48	4
1980	1989			1					1	46						13	1					no	62	5
1981	1990			1					1	61						13	1					no	77	5
1982	1991			1					1	73						12	1					no	88	5
1983	1992			1					1	78						10	1		2			no	93	6
1984	1993			1					1	75						10			4			no	91	5
1985	1994			1					1	79						10			10			no	101	5
1986	1995		2	1					1	75						12			11			no	102	6
1987	1996		3						1	70						10			14			no	98	5
1988	1997		5			41			29	65						10			14			no	164	6
1989	1998		9			66			45	54						10		2	44			no	230	7
1990	1999		8			81			60	60						10		2	53		15	yes	289	8
1991	2000		13			84			83	63						11		2	54	1	48	yes	359	9
1992	2001		18			89			113	63				2		11		2	95	1	67	yes	461	10
1993	2002		28			96			136	54				3		11		2	104	9	80	yes	523	10
1994	2003		27			130			146	57				3		10		2	111	11	91	yes	588	10
1995	2004		27			211			159	46				3		12		2	113	11	101	yes	685	10
1996	2005		30			221		10	144	40				3		19		2	122	13	105	yes	709	11
1997	2006		33			227		10	122	34				3		19		2	127	14	108	yes	699	11
1998	2007		26			234		10	120	33				3		19		2	134	14	114	yes	709	11
1999	2008		23			238		10	111	29		16		3		20	11	2	140	16	130	yes	749	13
2000	2009		20			244		10	113	22		16		3		24	29	2	137	34	159	yes	813	13
2001	2010		15			245		10	112	16	21	16		3		19	29	2	140	46	191	yes	865	14
2002	2011		9		1	245		10	126	8	44	16		3		20	40	2	136	46	231	yes	937	15
2003	2012		4		1	246		10	147	8	62	16		3		21	40	2	185	83	258	yes	1086	15
2004	2013		1		1	246		10	177	9	82	16		3	20	26	40	2	213	85	284	yes	1215	16
2005	2014		1		1	246		10	198	8	86	16		3	20	24	38	2	213	85	302	yes	1253	16
2006	2015		3	4	1	245	1	10	226	12	87	16	3	3	20	25	37	2	214	85	314	yes	1308	19
2007	2016	1	2	4		247	1	10	247	12	87	16	5	3	20	27	38	2	214	89	331	yes	1356	19
2008	2017	2	2	5		247	10	10	228	10	86	16	5	3	20	25	39	2	226	89	344	yes	1369	19
2009	2018	2	2	5		247	10	10	198	8	86	16	5	3	20	26	39	2	228	89	334	yes	1330	19
2010	2019	2	1	5		247	10		139	8	84	16	5	3	20	29	25	2	210	82	309	yes	1197	18
2011	2020	1	1	5		239	10		66	7	78	16	5		20	26	25	2	190	80	293	yes	1064	17
2012	2020	1	1	5		214	10		18	5	52	16	5		20	22	25	2	180	79	270	no	925	17
2013	2020	1	1	2		77	10		6	5	14	16	2			21	25	2	111	75	235	no	603	16
2014	2020			1		4	10		6	3	10	13	2			15	25	2	102	54	138	no	385	14
2015	2020																21					no	21	1
