# Supplementary material for: The recovery of European freshwater biodiversity has come to a halt
Source: Nature. 2023 Aug 9;620(7974):582–8. doi: 10.1038/s41586-023-06400-1 (PMC10432276; doi:10.1038/s41586-023-06400-1)
Supplement: Supplementary file 2 — Reporting Summary [file 41586_2023_6400_MOESM2_ESM.pdf]

nature portfolio

Corresponding author(s): Peter Haase

Last updated by author(s): Apr 24, 2023

Reporting Summary

Nature Portfolio wishes to improve the reproducibility of the work that we publish. This form provides structure for consistency and transparency in reporting. For further information on Nature Portfolio policies, see our Editorial Policies and the Editorial Policy Checklist.

Statistics

For all statistical analyses, confirm that the following items are present in the figure legend, table legend, main text, or Methods section.

n/a	Confirmed	
☐	☒	The exact sample size (*n*) for each experimental group/condition, given as a discrete number and unit of measurement
☐	☒	A statement on whether measurements were taken from distinct samples or whether the same sample was measured repeatedly
☐	☒	The statistical test(s) used AND whether they are one- or two-sided<br>*Only common tests should be described solely by name; describe more complex techniques in the Methods section.*
☐	☒	A description of all covariates tested
☐	☒	A description of any assumptions or corrections, such as tests of normality and adjustment for multiple comparisons
☐	☒	A full description of the statistical parameters including central tendency (e.g. means) or other basic estimates (e.g. regression coefficient) AND variation (e.g. standard deviation) or associated estimates of uncertainty (e.g. confidence intervals)
☐	☒	For null hypothesis testing, the test statistic (e.g. *F*, *t*, *r*) with confidence intervals, effect sizes, degrees of freedom and *P* value noted<br>*Give P values as exact values whenever suitable.*
☐	☒	For Bayesian analysis, information on the choice of priors and Markov chain Monte Carlo settings
☐	☒	For hierarchical and complex designs, identification of the appropriate level for tests and full reporting of outcomes
☒	☐	Estimates of effect sizes (e.g. Cohen's *d*, Pearson's *r*), indicating how they were calculated

Our web collection on statistics for biologists contains articles on many of the points above.

Software and code

Policy information about availability of computer code

Data collection	No software was used in data collection.
Data analysis	Annotated R (ver. 4.2.2); all scripts are available at GitHub: https://github.com/Ewelti/EuroAquaticMacroInverts

For manuscripts utilizing custom algorithms or software that are central to the research but not yet described in published literature, software must be made available to editors and reviewers. We strongly encourage code deposition in a community repository (e.g. GitHub). See the Nature Portfolio guidelines for submitting code & software for further information.

Data

Policy information about availability of data

All manuscripts must include a data availability statement. This statement should provide the following information, where applicable:

- Accession codes, unique identifiers, or web links for publicly available datasets
- A description of any restrictions on data availability
- For clinical datasets or third party data, please ensure that the statement adheres to our policy

Metadata, site characteristics, and trend estimates are available on GitHub: https://github.com/Ewelti/EuroAquaticMacroInverts. Raw biodiversity data will be made available in the same GitHub repository and to the BioTime database following acceptance and a six-month embargo.

Research involving human participants, their data, or biological material

Policy information about studies with human participants or human data. See also policy information about sex, gender (identity/presentation), and sexual orientation and race, ethnicity and racism.

Reporting on sex and gender	Does not apply to our study
Reporting on race, ethnicity, or other socially relevant groupings	Does not apply to our study
Population characteristics	Does not apply to our study
Recruitment	Does not apply to our study
Ethics oversight	Does not apply to our study

Note that full information on the approval of the study protocol must also be provided in the manuscript.

Field-specific reporting

Please select the one below that is the best fit for your research. If you are not sure, read the appropriate sections before making your selection.

☐ Life sciences ☐ Behavioural & social sciences ☒ Ecological, evolutionary & environmental sciences

For a reference copy of the document with all sections, see nature.com/documents/nr-reporting-summary-flat.pdf

Ecological, evolutionary & environmental sciences study design

All studies must disclose on these points even when the disclosure is negative.

Study description	The study is a meta-analysis of 1,816 time series of freshwater macroinvertebrate communities to examine biodiversity trends over time and across Europe. Overall estimates of slopes of biodiversity metrics were calculated using a Bayesian hierarchical model (2-step model). Step 1 involved calculating individual slopes for each time series. Step 2 involved an calculating overall estimate and an overall intercept and two random effects (country and study identity) as predictors.
Research sample	Data were collected from previous studies and assembled from a data call.
Sampling strategy	No sample-size calculation was preformed. Time series were included in analyses when they met selection criteria, resulting in a collection of 1,816 time series.
Data collection	Data were assembled from a data call to European ecologists and environmental managers. Peter Haase put out the data call and Ellen Welti assembled data from data providers.
Timing and spatial scale	All of the 1,816 time series contain annual sampling of a minimum of 8 years of data. All time series combined span the period of 1968-2020.
Data exclusions	All time series obtained in the data call were included if they met the pre-selected criteria of: 1) inclusion of abundance estimates, 2) surveyed whole freshwater invertebrate communities (not restricted to certain taxonomic groups, such as insects), 3) identified most major taxa to family, genus or species, 4) had a minimum of eight sampling years (not necessarily consecutive), 5) had no changes in sampling method or taxonomic resolution during the sampling period, and 6) had consistent sampling effort per site (e.g. number of samples or area of river sampled) across years.
Reproducibility	No new experiments were performed in this meta-analysis. All code, meta-data, and slope estimates are provided on Github: https://github.com/Ewelti/EuroAquaticMacroInverts
Randomization	The study is a meta-analysis of pre-collected time series data, and does not including new experimental designs requiring randomization. When testing for overall estimates of change in biodiversity metrics over time, study and country were included in models as random effects.
Blinding	Blinding was not relevant to this study.

Did the study involve field work? ☐ Yes ☒ No

Reporting for specific materials, systems and methods

We require information from authors about some types of materials, experimental systems and methods used in many studies. Here, indicate whether each material, system or method listed is relevant to your study. If you are not sure if a list item applies to your research, read the appropriate section before selecting a response.

Materials & experimental systems

n/a	Involved in the study
☒	☐ Antibodies
☒	☐ Eukaryotic cell lines
☒	☐ Palaeontology and archaeology
☐	☒ Animals and other organisms
☒	☐ Clinical data
☒	☐ Dual use research of concern
☒	☐ Plants

Methods

n/a	Involved in the study
☒	☐ ChIP-seq
☒	☐ Flow cytometry
☒	☐ MRI-based neuroimaging

Animals and other research organisms

Policy information about studies involving animals; ARRIVE guidelines recommended for reporting animal research, and Sex and Gender in Research

Laboratory animals	The study did not involve laboratory organisms.
Wild animals	Data include time series from previous studies of field collections of freshwater macroinvertebrates. Macroinvertebrates were killed to identify specimens in these studies. Details are provided in the Methods and Supplemental Tables.
Reporting on sex	Does not apply to our study.
Field-collected samples	Data include time series from previous studies of field collections of freshwater macroinvertebrates. Macroinvertebrates were killed to identify specimens in these studies. Details are provided in the Methods and Supplemental Tables.
Ethics oversight	No ethical approval or guidance was required as data were collected only from previous studies.

Note that full information on the approval of the study protocol must also be provided in the manuscript.
